# Transosseous Suture Fixation of an Inverted Palatal Mucoperiosteal Flap to Residual Buccal Alveolar Bone for Oronasal Fistula Repair in a Dog

**DOI:** 10.3390/vetsci13101097

**Published:** 2026-10-15

**Authors:** Jeong-Heon Lee, Sangyul Lee, Jun-Sik Cho, Changmin Park, Beom Joon Park, Seohyeon Yoon, Lim Jeongwon, Jaehwan Kim, Hwi-Yool Kim, Jung-Moon Kim

**Affiliations:** 1 Department of Veterinary Surgery, College of Veterinary Medicine, Konkuk University, 120 Neungdong-ro, Gwangjin-gu, Seoul 05029, Republic of Korea; jim3224@konkuk.ac.kr (J.-H.L.); sangyul7918@konkuk.ac.kr (S.L.); cmpark9575@konkuk.ac.kr (C.P.); park0510k@konkuk.ac.kr (B.J.P.); hykim@konkuk.ac.kr (H.-Y.K.); 2 Department of Veterinary Radiology, College of Veterinary Medicine, Konkuk University, 120 Neungdong-ro, Gwangjin-gu, Seoul 05029, Republic of Korealimjw2002@konkuk.ac.kr (L.J.); jaehwan@konkuk.ac.kr (J.K.)

**Keywords:** dog, periodontal disease, double-layer closure, oral reconstructive surgery, computed tomography, long-term follow-up

## Abstract

Advanced periodontal disease can compromise the recipient tissues available for flap fixation during oronasal fistula repair. This report describes double-layer repair of an acquired periodontal oronasal fistula in an eight-year-old castrated male Beagle. Radiographs and computed tomography (CT) identified regional bone loss and residual alveolar bone adjacent to the defect. Following extraction of the left maxillary canine and second premolar and debridement of the fistula margins, an inverted hard palatal mucoperiosteal flap was anchored to residual buccal alveolar bone through four transosseous bone holes created with an 18-gauge needle. Transosseous suture fixation used 4-0 polydioxanone simple interrupted sutures. A buccal mucosal advancement flap covered the inverted flap and palatal donor site; no interpositional graft was used. Healing without clinically evident dehiscence was documented on postoperative day 18, and nasal discharge had resolved by day 45. At 555 days, visual oral examination revealed an intact repair, with concordant CT findings obtained for an unrelated otologic indication. This case illustrates the use of residual buccal alveolar bone for palatal flap attachment when periodontal tissue loss limits gingival recipient margins, with maintained closure documented at late clinical and imaging reassessment.

## Simple Summary

Advanced periodontal disease can create an abnormal opening between the mouth and nasal cavity in dogs. Closing this opening may be difficult when both the bone and the surrounding soft tissues have been damaged. This report describes an eight-year-old Beagle treated using two local tissue flaps. A flap raised from the hard palate was turned over the opening palatal to the left maxillary canine tooth and attached to the remaining buccal alveolar bone using four sutures passed through small bone holes. A second flap was advanced from the cheek side to cover the repair. Bone suture fixation provided attachment points where the soft tissue was considered unreliable for fixation. The dog began eating voluntarily on the day after surgery and was discharged four days after surgery. Healing was documented at 18 days, nasal discharge resolved, and no recurrence was recorded during 45 days of follow-up. At 555 days after surgery, examination of the mouth showed an intact repair, and a computed tomography scan performed for an unrelated ear condition also supported maintained closure. This case illustrates how the available bone and soft tissues can be incorporated into planning a two-layer fistula repair. Additional cases are needed to determine the benefits and limitations of this fixation method.

## 1. Introduction

Acquired oronasal fistulas are epithelialized communications between the oral and nasal cavities, and periodontal disease is a common underlying cause in dogs. Progressive periodontal destruction on the palatal aspect of the maxillary canine tooth can compromise the adjacent alveolar partition and establish communication with the nasal cavity [1,2,3]. Secondary nasal inflammation may result in persistent nasal discharge and sneezing. Treatment therefore combines management of the affected teeth and periodontal tissues with surgical restoration of a barrier between the oral and nasal cavities [4,5,6].

Local flap reconstruction requires consideration of defect location and extent, available donor tissue, and the condition of the recipient margins [6,7]. Preservation of vascular supply, careful tissue handling, and apposition without excessive tension are central to healing [6,8]. Double-flap repair has been described for canine oronasal fistulas [9,10]. In a configuration used for recurrent periodontal defects, an inverted palatal mucoperiosteal flap provides the nasal-side layer, with its epithelial surface facing the nasal cavity, while an overlying buccal advancement flap provides oral coverage [9]. Coverage of the exposed palatal donor site must also be considered when planning buccal flap advancement [11].

Securing the nasal-side flap presents an additional challenge when periodontal destruction compromises the buccal gingival recipient margins. Residual bone at the defect periphery may provide suture attachment sites despite loss of the osseous partition beneath the fistula. Fixation of inverted palatal tissue through surgically created bone holes, followed by mucogingival coverage of the fistula and donor site, has been described [11]. Transosseous suture fixation has also been used to secure the trailing edge of a palatal transposition flap to palatal bone following treatment of nasal transmissible venereal tumor [12]. These precedents provide a rationale for considering this fixation method alongside flap coverage when suitable soft-tissue attachment sites are limited.

The present report describes double-layer repair of an acquired oronasal fistula associated with advanced periodontal disease in a dog, using transosseous suture fixation of an inverted palatal mucoperiosteal flap to residual buccal alveolar bone. It details the recipient anatomy, selection of four fixation sites, and sequence of flap apposition and fixation, together with the clinical and computed tomographic findings at late postoperative reassessment.

## 2. Case Description

### 2.1. Signalment, History, and Clinical Findings

An eight-year-old castrated male Beagle was evaluated at Konkuk University Veterinary Teaching Hospital for an acquired oronasal fistula associated with advanced periodontal disease. The dog had recently been hospitalized for seizure-like episodes and suspected systemic inflammatory response syndrome and had been medically stabilized before consultation with the surgical service. At the initial oral assessment, the dog weighed 9.13 kg and had a body condition score of 5/9. Blood-tinged nasal discharge was observed from the left nostril.

**Figure 1:**
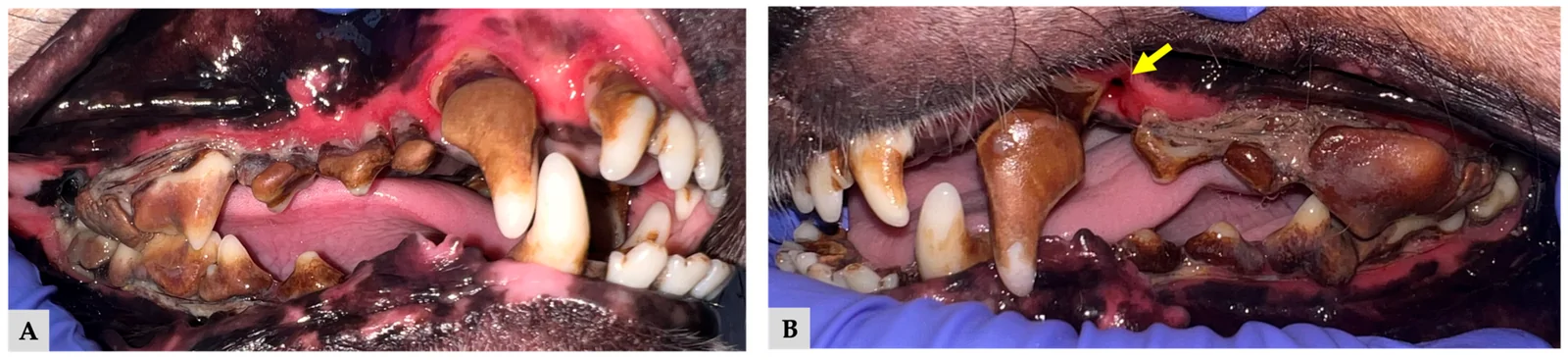
Preoperative oral findings in a dog with advanced periodontal disease. (**A**) Right lateral oral view. (**B**) Left lateral oral view. Both views show extensive dental calculus, marked gingival erythema, and gingival recession. In panel (**B**), the yellow arrow indicates an oronasal fistula adjacent to the left maxillary canine tooth (204).

Oral examination revealed extensive, thick calculus deposits covering more than two-thirds of the crowns of affected teeth, marked gingival erythema and edema, gingival recession, root exposure, and bleeding on manipulation (Figure 1). Periodontal assessment documented more than 50% loss of periodontal attachment and alveolar support at severely affected teeth, consistent with stage 4 periodontal disease.

The left maxillary canine tooth, designated 204 according to the modified Triadan system, exhibited grade 3 mobility. The left maxillary second premolar tooth (206) was also markedly mobile, and the left maxillary first premolar tooth (205) was already absent at presentation. An oronasal fistula was identified on the palatal aspect of tooth 204 and extended toward the region of the missing tooth 205. Communication between the oral and nasal cavities was confirmed by catheter passage through the fistula. Bilateral nasal patency was documented. No previous surgical attempt to close the fistula was recorded.

### 2.2. Diagnostic Evaluation and Preoperative Preparation

#### 2.2.1. Diagnostic Imaging

**Figure 2:**
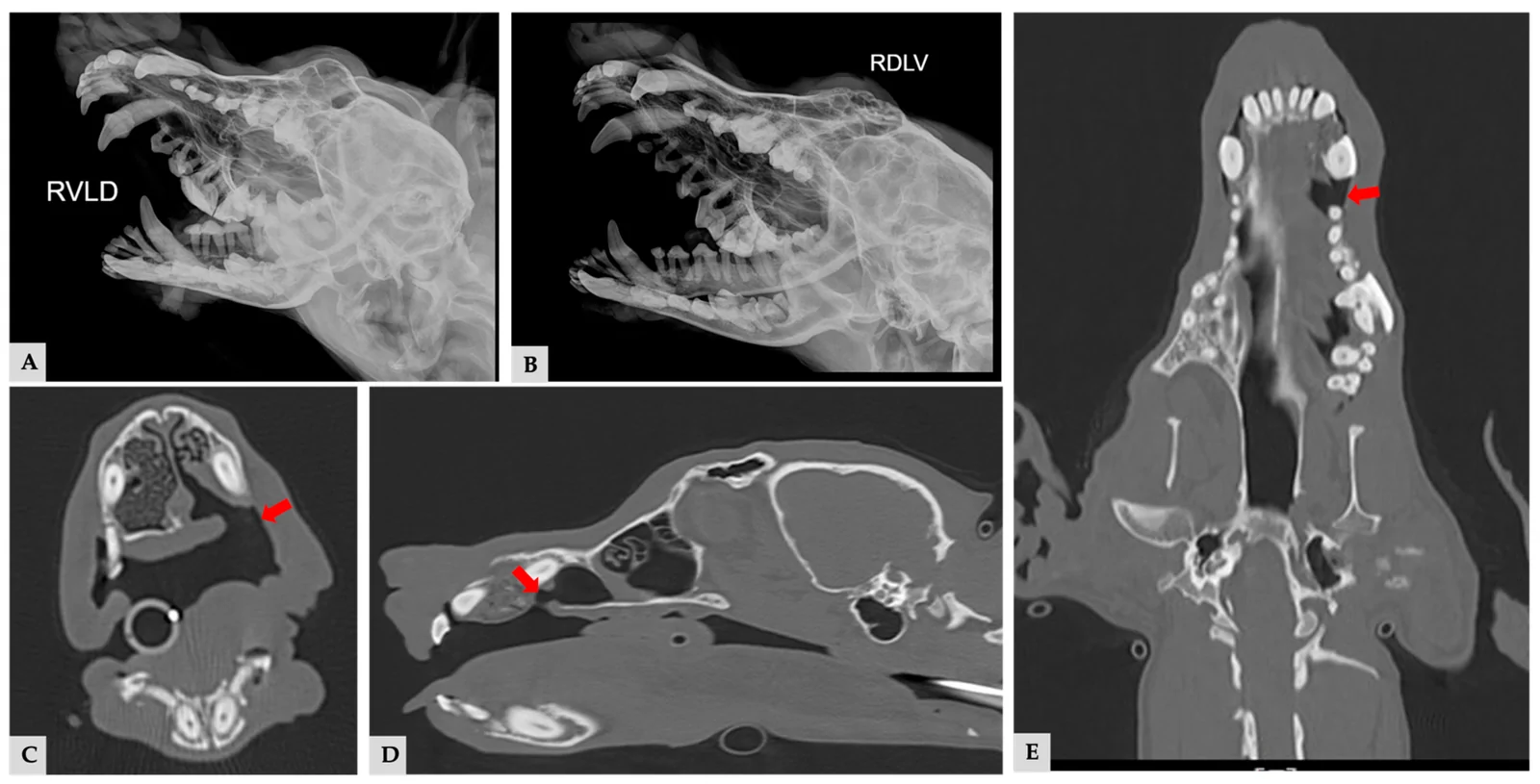
Preoperative radiographic and computed tomographic findings. (**A**,**B**) Oblique skull radiographs showing generalized horizontal alveolar bone loss and reduced alveolar support around multiple teeth. (**C**–**E**) Transverse (**C**), sagittal (**D**), and coronal (**E**) computed tomography images demonstrating alveolar and adjacent palatal bone loss in the left maxillary canine region. Red arrows indicate the osseous defect at the oronasal fistula site, where the oral cavity communicates with the left nasal cavity. Associated fluid- to soft-tissue-attenuating material and turbinate bone loss are evident within the affected nasal cavity.

Diagnostic imaging included skull radiography and computed tomography (CT) (Figure 2). Radiographs demonstrated generalized horizontal alveolar bone loss affecting the maxilla and mandible, with loss of alveolar support at the furcations of multiple teeth. The absence of tooth 205 was also confirmed.

CT was performed before dental scaling using a CT scanner (Aquilion Lightning; Canon Medical Systems, Otawara, Japan). It demonstrated alveolar and adjacent palatal osteolysis around the root of tooth 204, with communication between the oral cavity and the left nasal cavity. Fluid- to soft-tissue-attenuating material accumulated within the affected nasal cavity, accompanied by loss of turbinate bone. CT also demonstrated generalized alveolar bone loss and mineral-attenuating deposits on the external surfaces of multiple teeth, corresponding to the periodontal disease and extensive calculus observed clinically.

**Figure 3:**
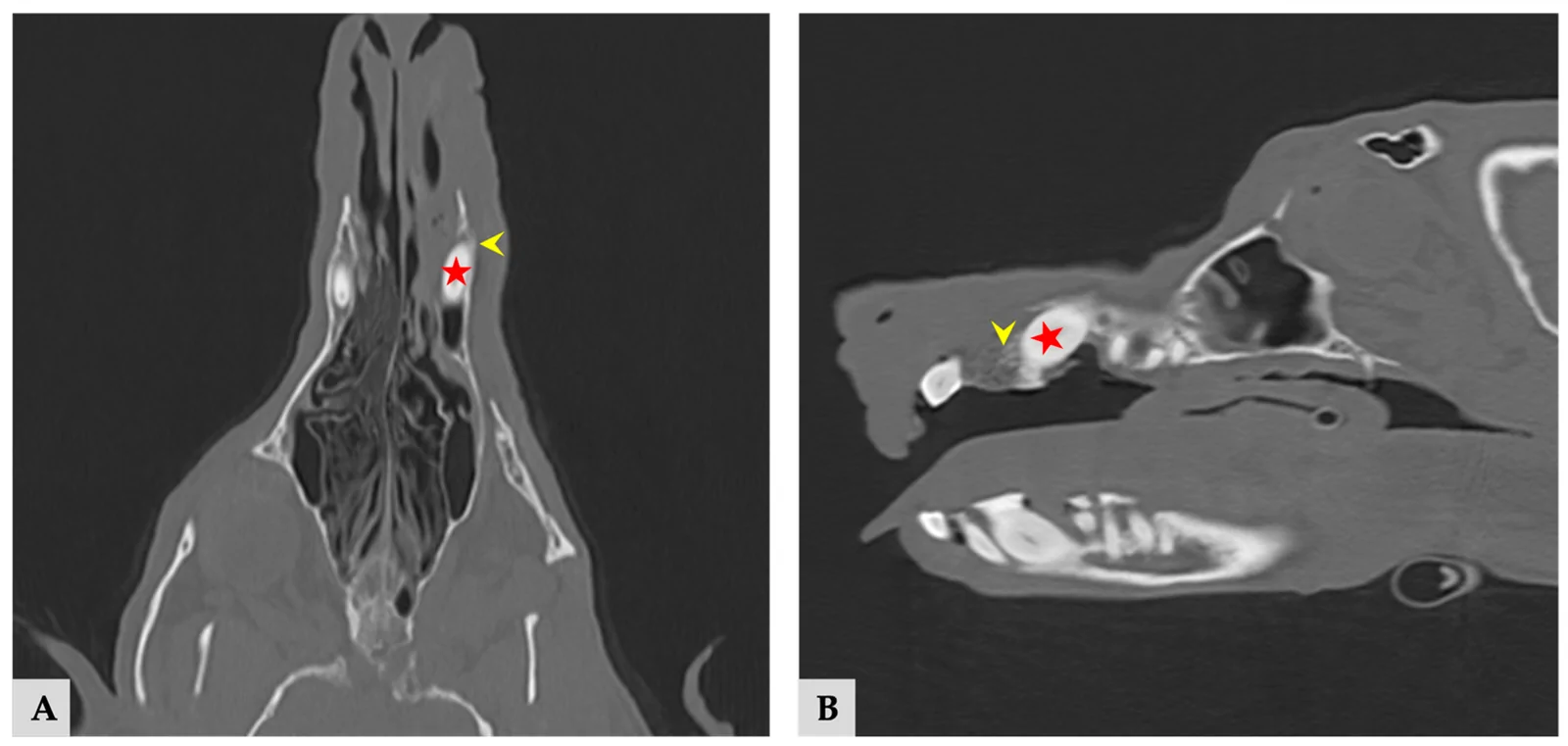
Preoperative computed tomographic appearance of residual buccal alveolar bone. (**A**) Coronal view. (**B**) Sagittal view. Red asterisks identify the left maxillary canine tooth (204), which was subsequently extracted. Yellow arrowheads indicate residual buccal alveolar bone approximately corresponding to the rostral fixation region used during reconstruction. Individual transosseous suture sites were selected by direct intraoperative inspection after tooth extraction and debridement.

The oral examination and dentoalveolar imaging findings supported a diagnosis of an acquired oronasal fistula associated with advanced periodontal disease. Residual alveolar bone was visible adjacent to the defect on qualitative assessment of the radiographs and CT images (Figure 3). Quantitative measurements of the residual bone at individual potential suture attachment sites were not available.

#### 2.2.2. Dental Preparation and Preoperative Assessment

**Figure 4:**
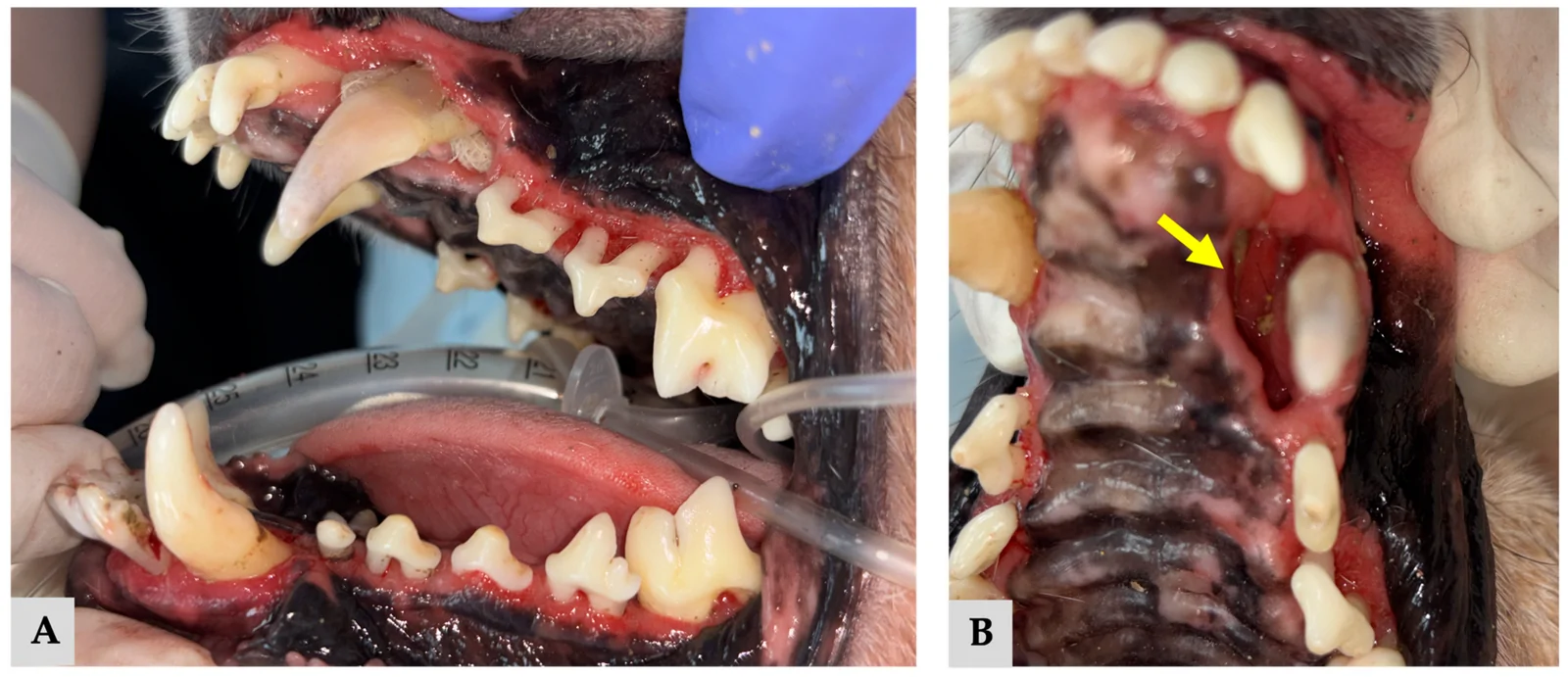
Oral findings after dental scaling and before tooth extraction. (**A**) Left lateral oral view showing marked gingival erythema and recession with root exposure following calculus removal. (**B**) Ventral view of the hard palate showing the oronasal fistula on the palatal aspect of the left maxillary canine tooth (204; yellow arrow).

Dental scaling was performed on 31 January 2025 without tooth extraction. Removal of the extensive calculus exposed markedly recessed gingival margins and inflamed periodontal tissues that bled readily during manipulation (Figure 4). Following scaling, the fistula measured approximately 22 mm × 8 mm (length x width). This measurement represented the opening before extraction of the adjacent teeth and surgical preparation of the defect.

Definitive reconstruction was performed on 24 February 2025, 24 days after scaling. At the preoperative assessment, the dog weighed 10.0 kg and had a body condition score of 5/9. Laboratory testing on the day of surgery showed a white blood cell count of 8.66 × 10^3^/µL, a packed cell volume of 43%, a platelet count of 364 × 10^3^/µL, and a serum albumin concentration of 2.8 g/dL. Serum C-reactive protein (CRP) was elevated at 2.9 mg/dL relative to the laboratory reference interval of 0.1–1.0 mg/dL.

### 2.3. Surgical Planning and Anesthesia

For this initial repair, the surgeon prioritized obtaining a secure closure, considering the possibility that additional tissue loss and scarring after failure could complicate subsequent reconstruction. A double-layer reconstruction was planned using an inverted palatal mucoperiosteal flap for the nasal-side layer and a buccal mucosal advancement flap for oral coverage. Documented gingival recession and periodontal tissue loss raised concern about attaching the free margin of the inverted flap to the buccal gingival margin alone. Transosseous sutures were therefore planned to maintain flap apposition using residual buccal alveolar bone as an alternative attachment site. The buccal flap was intended to cover both the inner repair and the exposed palatal donor site. Imaging informed the anticipated availability of bone, while final attachment-site selection and assessment of flap reach were reserved for intraoperative evaluation after extraction and defect preparation.

Premedication consisted of butorphanol (Butophan Inj.; Myungmoon Pharm, Seoul, Republic of Korea) at 0.2 mg/kg intravenously, followed by midazolam (Bukwang Midazolam Inj., Bukwang Pharm, Seoul, Republic of Korea) at 0.2 mg/kg intravenously. Maropitant (Cerenia, Zoetis, Parsippany, NJ, USA) was administered at 1 mg/kg subcutaneously. Anesthesia was induced with propofol (Anepol Inj., Hana Pharm, Seoul, Republic of Korea) at 3 mg/kg intravenously and maintained with isoflurane (Kyongbo Pharm, Asan, Republic of Korea). Intraoperative analgesia included a butorphanol constant-rate infusion at 0.2 mg/kg/h. Amoxicillin–clavulanate (Amocla, Kuhnil Pharmaceutical, Seoul, Republic of Korea) was administered perioperatively at 13.75 mg/kg intravenously.

### 2.4. Surgical Procedure

#### 2.4.1. Tooth Extraction and Defect Preparation

The owner was advised of the need for further periodontal assessment and possible additional extractions. At the owner’s request, extractions during fistula repair were limited to teeth 204 and 206. Teeth 204 and 206 were extracted, and the epithelialized fistula margins were debrided to prepare the recipient tissues. The dimensions of the final defect after extraction and debridement were not available. The residual buccal alveolar bone was directly inspected, and sites considered suitable for transosseous suture placement were selected.

#### 2.4.2. Transosseous Suture Fixation of the Palatal Flap

**Figure 5:**
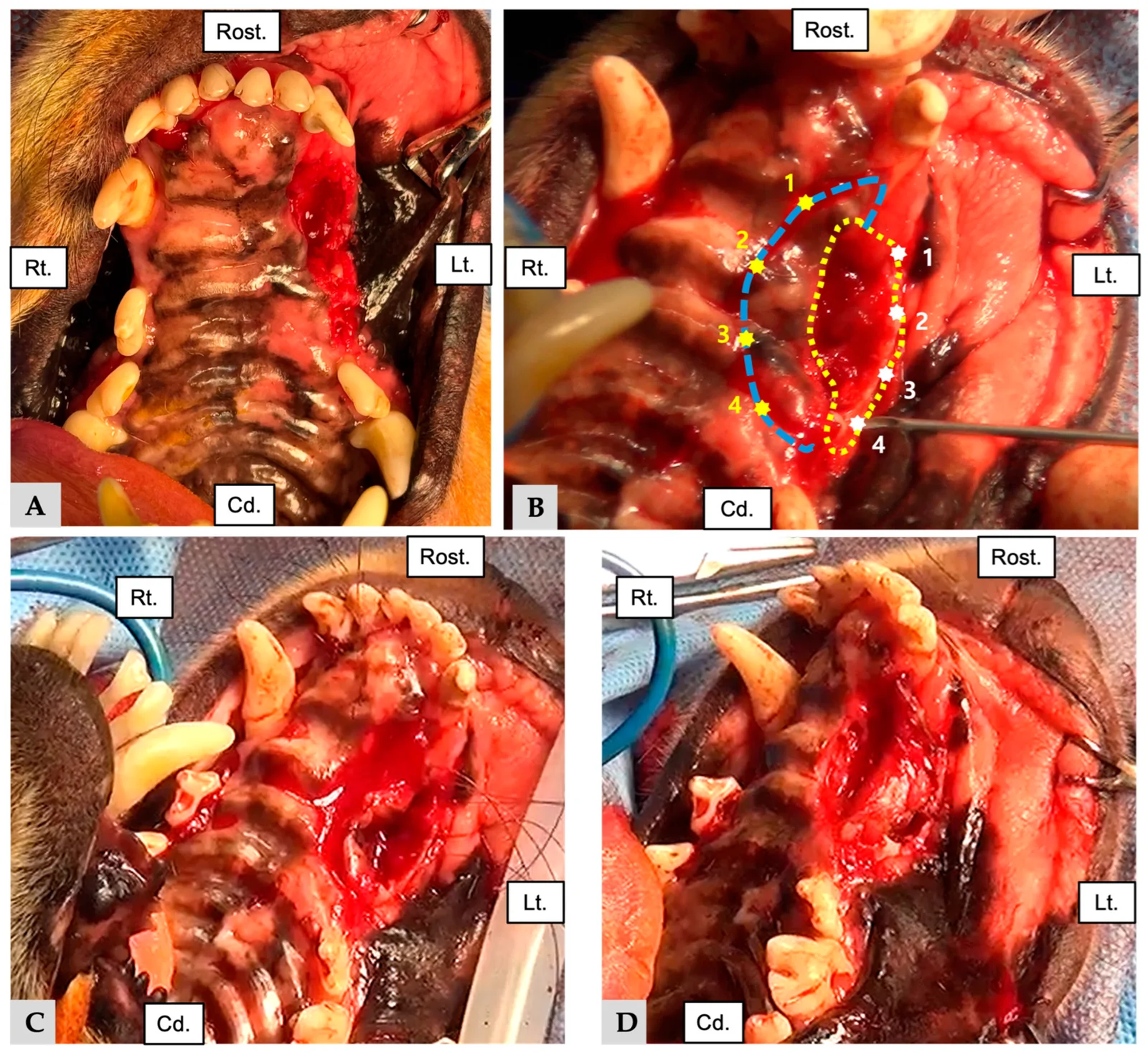
Palatal flap preparation and transosseous suture fixation of the nasal-side layer. Panels (**A**,**B**) show ventral views of the hard palate; panels (**C**,**D**) show oblique ventral views obtained from the dog’s left side. (**A**) Appearance of the defect following extraction of teeth 204 and 206 and preparation of the fistula margins. (**B**) The blue dashed line delineates the palatal flap margin before inversion, and the yellow dotted line indicates its intended position over the defect. Yellow asterisks and numbers (1–4) mark the flap suture sites, and white asterisks and numbers (1–4) mark the corresponding sites for transosseous bone holes in the residual buccal alveolar bone. Sites bearing the same number were connected by a single transosseous suture. (**C**) Four preplaced, untied 4-0 polydioxanone sutures. Gentle traction on the sutures allowed provisional positioning of the flap and assessment of its reach, tension, and apposition before tying. (**D**) Inverted palatal flap secured with four simple interrupted transosseous sutures before buccal flap advancement. Rost., rostral; Cd., caudal; Rt., right; Lt., left.

A palatal mucoperiosteal flap was designed medial to the defect and larger than the opening to allow coverage without excessive tension. Using a No. 15 scalpel blade, a curvilinear incision was made from a point slightly caudomedial to the left maxillary third incisor (203) to a point rostromedial to the left maxillary third premolar (207). The flap was elevated from its medial aspect toward the defect using a periosteal elevator, while retaining its attachment along the palatal margin of the fistula. Hemorrhage was controlled with gauze pressure. The flap was then inverted laterally over the opening, positioning its original oral epithelial surface toward the nasal cavity (Figure 5).

**Figure 6:**
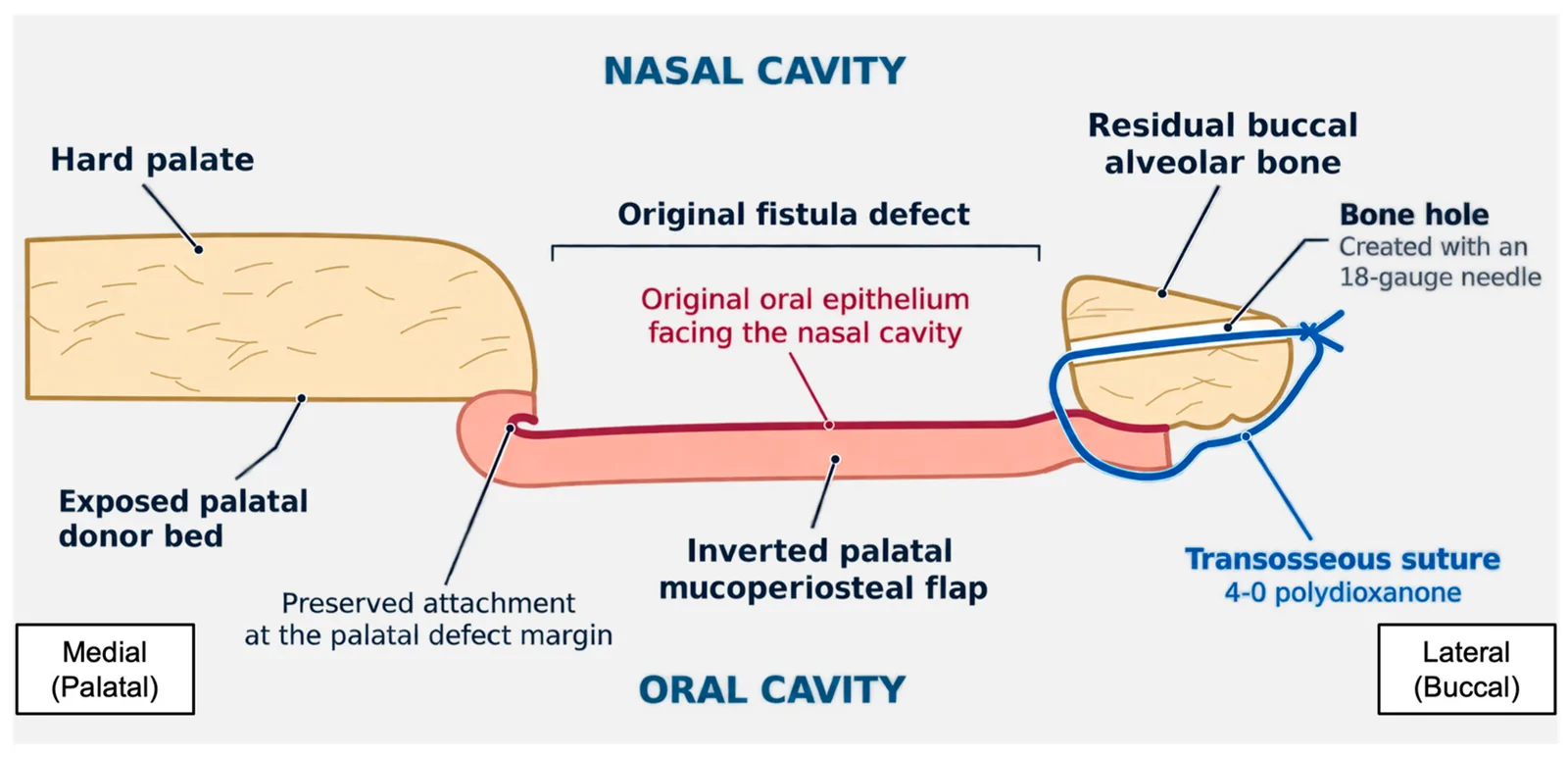
Schematic cross-section of transosseous suture fixation of the inverted palatal flap.

Four transosseous bone holes were created in the residual buccal alveolar bone using an 18-gauge needle (Sungshim Medical, Bucheon, Republic of Korea) introduced from the lateral aspect and directed medially. The rostralmost bone hole was positioned slightly caudal to the rostral margin of the defect, and the caudalmost bone hole was positioned slightly rostral to its caudal margin. Two additional bone holes were placed at approximately even intervals between these sites, providing four attachment points along the residual buccal alveolar bone. The bone-hole positions were selected to correspond to the free margin of the inverted flap and permit apposition without excessive traction. At each attachment site, the suture needle carrying 4-0 polydioxanone (PDS; Ethicon, Somerville, NJ, USA) was passed through the preformed bone hole and then through the corresponding margin of the inverted palatal flap (Figure 6). All four sutures were initially left untied and gently drawn to provisionally position the flap at the intended attachment sites. This allowed assessment of flap reach, tension, and apposition at the planned attachment sites, while checking for focal puckering. Further mobilization with a periosteal elevator was performed to obtain satisfactory apposition before the sutures were individually tied in a simple interrupted pattern. The palatal flap was secured with these four transosseous sutures alone before advancement of the buccal flap.

The diagram illustrates the relationship between the inverted palatal mucoperiosteal flap, the original fistula defect, and the residual buccal alveolar bone at the stage shown in Figure 5D, before buccal flap advancement. One representative transosseous suture is shown passing through a preformed bone hole in the residual buccal alveolar bone and the free margin of the flap. The original oral epithelial surface of the inverted flap faces the nasal cavity. Not to scale.

#### 2.4.3. Buccal Mucosal Advancement and Final Closure

A buccal mucosal advancement flap was prepared to provide oral coverage. Two releasing incisions were made in the buccal tissues at approximately the levels of the rostral and caudal ends of the palatal flap. Both incisions extended away from the defect, opposite to the intended direction of flap advancement. The flap included buccal mucosa and underlying soft tissue, without incorporation of the periosteum. The flap was mobilized by undermining, and its reach and tension were assessed during medial advancement. It was then positioned to cover both the inverted palatal flap and the entire exposed palatal donor site.

**Figure 7:**
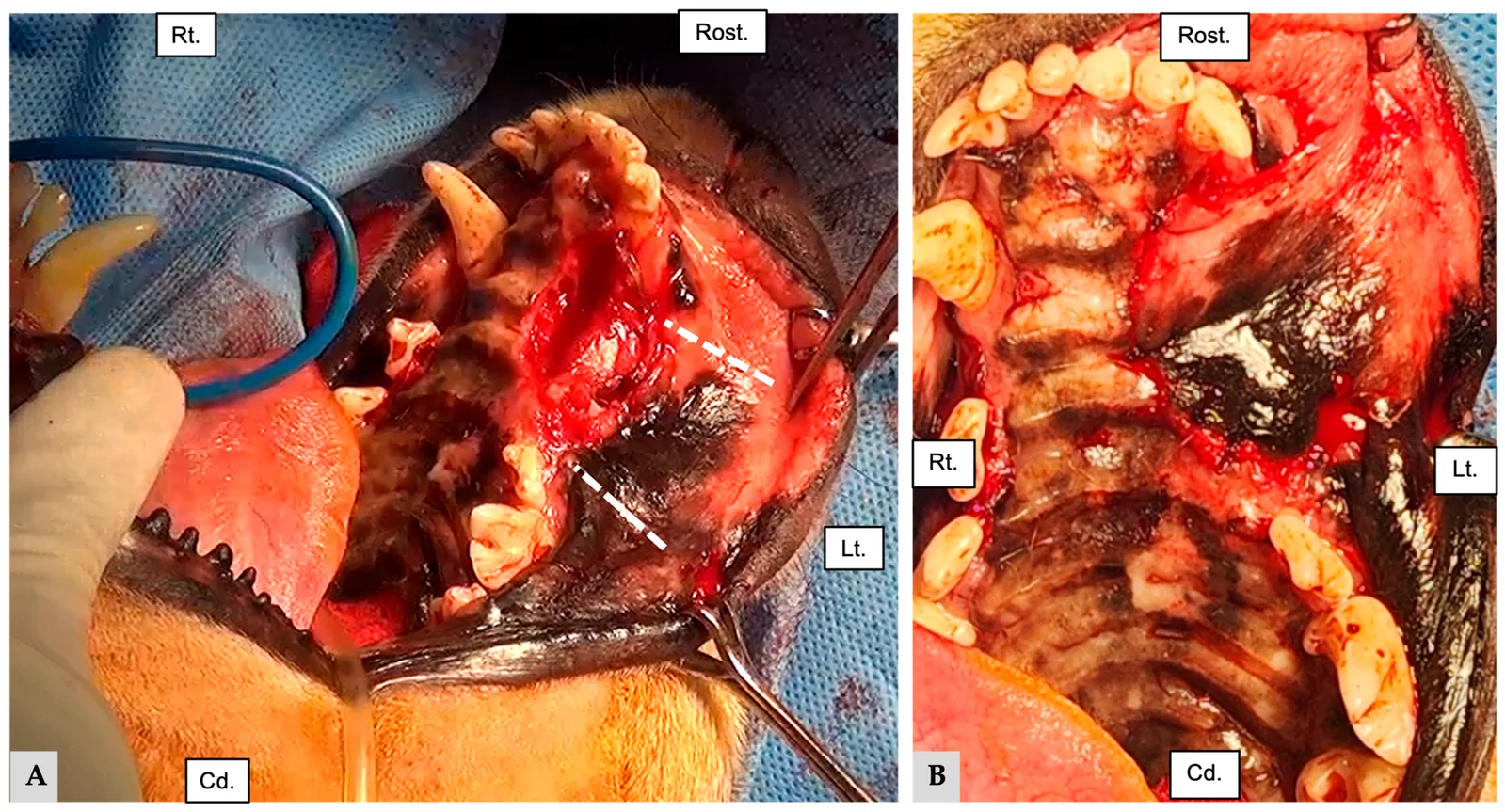
Buccal flap advancement and completion of double-layer closure. (**A**) Oblique ventral view showing the rostral and caudal releasing incisions used to mobilize the buccal mucosal advancement flap (white dashed lines). (**B**) Ventral view of the completed oral closure after advancement and suturing of the buccal flap over the inverted palatal flap and the exposed palatal donor site. Rost., rostral; Cd., caudal; Rt., right; Lt., left.

The buccal flap was secured to the surrounding recipient soft tissues using 4-0 polydioxanone simple interrupted sutures placed approximately 2 mm apart. Advancement positioned the final oral suture line away from the original fistula opening (Figure 7). Complete coverage was confirmed by direct inspection, and closure was assessed intraoperatively as tension-free. No cartilage graft, barrier membrane, or other interpositional graft was used. No immediate surgical complication was recorded.

### 2.5. Postoperative Management and Outcome

The dog remained hospitalized until postoperative day (POD) 4. On POD 1, intravenous crystalloid support was provided with Plasma-Lyte OP (JW Life Science, Dangjin, Republic of Korea) at a recorded total fluid administration rate of 2.5 mL/kg/h. Early postoperative analgesia comprised tramadol (Tridol; Yuhan Corporation, Seoul, Republic of Korea) at 10 mg/kg orally every 8 h on POD 1–2 and meloxicam (Metacam; Boehringer Ingelheim, Ingelheim am Rhein, Germany) at 0.1 mg/kg every 24 h, administered intravenously on POD 1 and subcutaneously on POD 2. Analgesic treatment was discontinued after POD 2 based on the attending clinician’s assessment of postoperative pain. Amoxicillin–clavulanate at 13.75 mg/kg and famotidine at 0.5 mg/kg were administered every 12 h, intravenously on POD 1–2 and orally on POD 3. Anticonvulsant treatment included oral phenobarbital at 2 mg/kg every 12 h on POD 1–2, with the dose reduced to 1.5 mg/kg every 12 h on POD 3. Potassium bromide was also administered orally at 10 mg/kg on POD 1. Concurrent oral medications on POD 1–3 included ursodeoxycholic acid at 7.5 mg/kg every 12 h and silymarin at 10 mg/kg every 12 h.

During hospitalization, the surgical site was assessed daily, local oral care was performed with 0.1% povidone–iodine, and a liquid recovery diet (Royal Canin Recovery) was provided. On POD 1, the surgical site showed moderate erythema, swelling, and warmth, with mild pain and blood-tinged discharge from the left nostril. These findings were consistent with expected early postoperative changes and were not classified as surgical complications. The dog voluntarily consumed the recovery diet. Recorded intake increased from approximately 1.0 times the resting energy requirement on POD 1 to 1.2 times on POD 2 and 1.5 times on POD 3–4. Local inflammation decreased to mild by POD 2, and nasal discharge gradually diminished. Daily examinations documented intact sutures without clinically evident dehiscence.

Between POD 1 and POD 4, the white blood cell count decreased from 17.88 to 5.64 × 10^3^/µL, and serum CRP declined from 6.9 to 2.5 mg/dL, although CRP remained above the laboratory reference interval. These trends, together with improving local signs, maintained wound closure, and adequate oral intake, supported discharge on POD 4. Mild local inflammation and diminishing blood-tinged nasal discharge were still present at discharge. A 5-day supply of amoxicillin–clavulanate at 13.75 mg/kg, famotidine at 0.5 mg/kg, phenobarbital at 1.5 mg/kg, ursodeoxycholic acid at 7.5 mg/kg, and silymarin at 10 mg/kg was prescribed, with instructions for oral administration every 12 h. The owner was instructed to provide only a liquid diet, avoid manipulating or brushing the surgical site, prevent chewing on toys or other objects, and use an Elizabethan collar. These measures were to be maintained until re-examination on POD 18.

**Figure 8:**
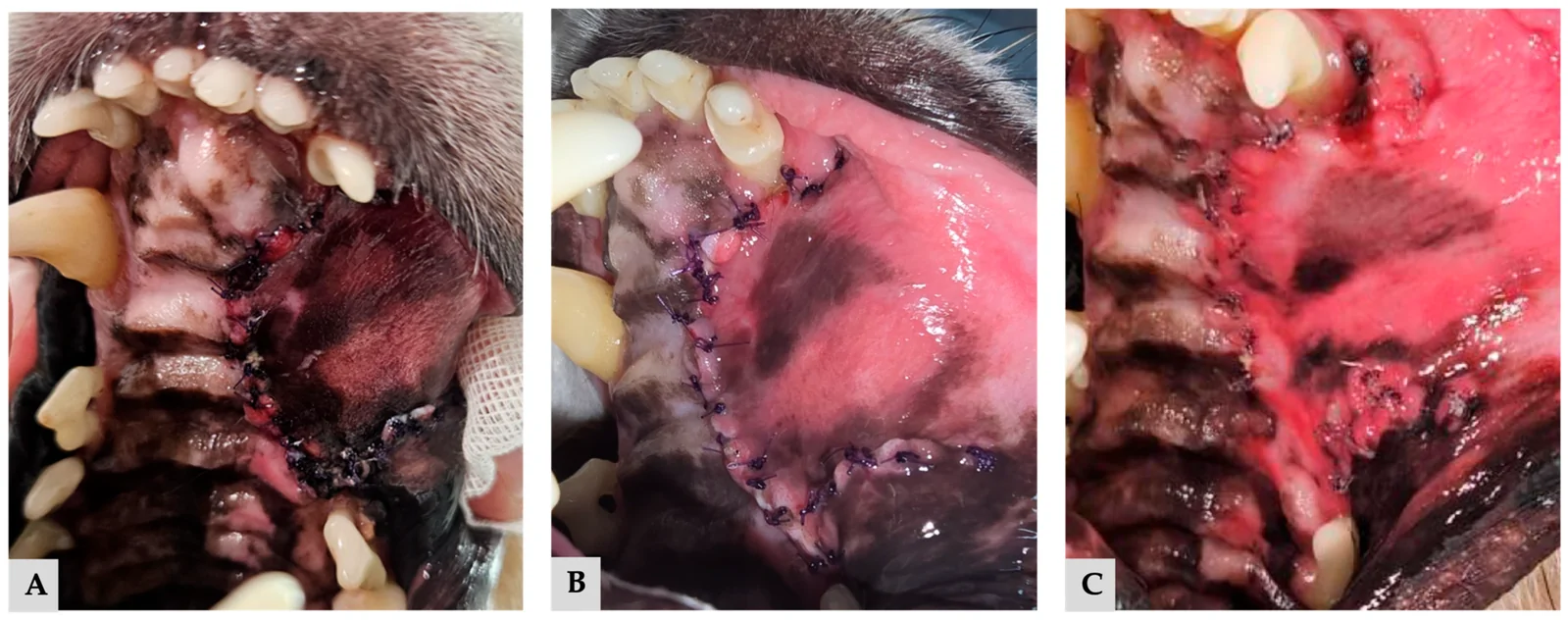
Postoperative appearance of the oronasal fistula repair. (**A**) POD 1, showing local erythema and swelling with sutures in place. (**B**) POD 4, on the day of discharge, showing maintained flap apposition and intact sutures. (**C**) POD 18 follow-up, showing healing of the repair without clinically evident dehiscence or recurrent fistulation.

Clinical examination and photographs on POD 18 documented healing without clinically evident dehiscence or recurrent fistulation (Figure 8). At an in-person re-examination on POD 45, owner interview, physical examination, and visual oral examination documented resolution of nasal discharge, maintained oral intake, and no clinically evident recurrent fistula or dehiscence.

**Figure 9:**
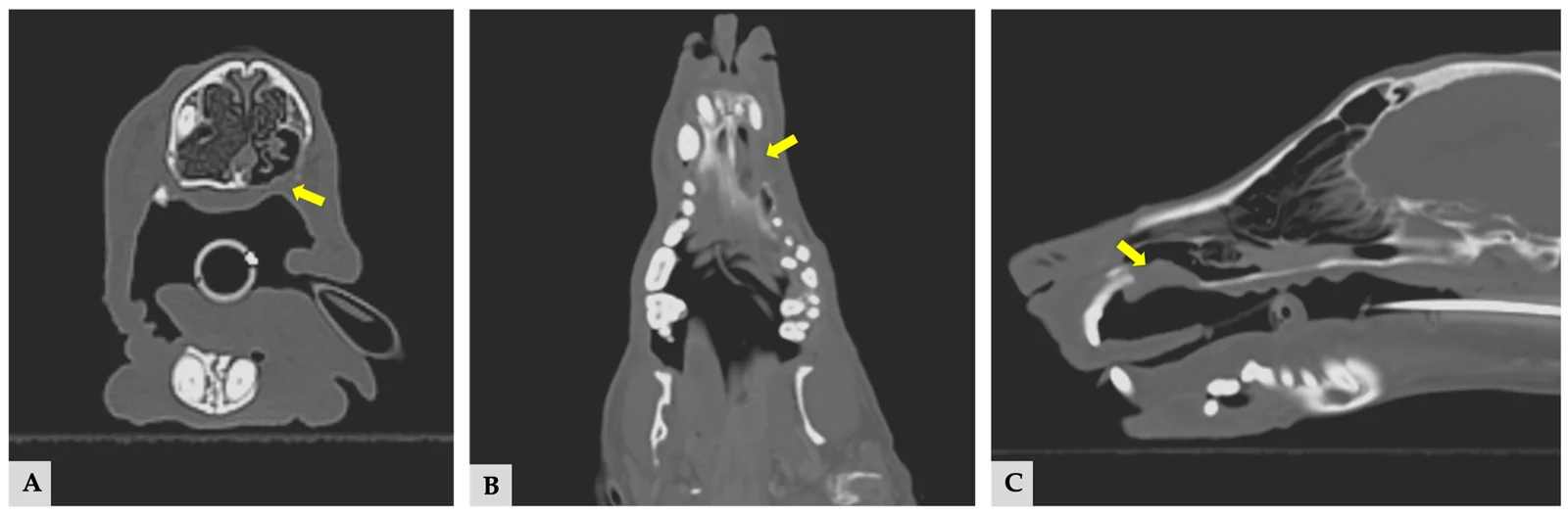
Postoperative computed tomographic assessment of the oronasal fistula repair. Transverse (**A**), coronal (**B**), and sagittal (**C**) images obtained 555 days after reconstruction at anatomical levels corresponding to the preoperative CT images in Figure 2. Yellow arrows indicate soft-tissue coverage at the site of the previous oronasal communication, consistent with maintained closure following flap reconstruction.

On 2 September 2026, 555 days after reconstruction, the dog underwent CT for an unrelated otologic indication. During the same visit, visual oral examination showed an intact repair without clinically evident dehiscence or recurrent fistulation. CT evaluation was also consistent with maintained closure at the previous fistula site (Figure 9).

## 3. Discussion

This case documents double-layer repair of a periodontal oronasal fistula in which compromised gingival recipient margins coexisted with usable local flap tissue and residual buccal alveolar bone. Transosseous sutures secured an inverted palatal mucoperiosteal flap, and a buccal advancement flap provided oral coverage. Oral examination and CT at 555 days were consistent with maintained closure. These observations illustrate how the condition of the recipient margins and the availability of adjacent tissues can be considered together when planning reconstruction.

Double-flap repair has previously been applied to canine oronasal fistulas [9,10]. An early clinical report described repair of recurrent periodontal fistulas with an inverted palatal flap and an overlying buccal advancement flap. The inverted flap was sutured to palatal and gingival tissues rostrally and caudally and to periosteal remnants along the buccal margin. The technique was also used for initial fistula repair in dogs considered at increased risk of failure with conventional closure [9]. Transosseous suture fixation has additionally been reported following treatment of nasal transmissible venereal tumor. In that case, the trailing edge of a palatal transposition flap was secured through holes in the palatal donor bone because suitable soft tissue for attachment was absent, and the donor site was left to heal by second intention [12].

Use of residual bone for inner-flap fixation had to be considered together with palatal flap reach and the extent of buccal flap coverage. Fixation of inverted palatal tissue through bone holes, followed by mucogingival coverage of both the fistula and the palatal donor site, was previously described by Legendre [11]. In the present dog, the attachment sites were selected to correspond to the free margin of the inverted flap, and provisional positioning allowed its reach and apposition to be assessed before tying. The buccal flap also had to cover the additional area exposed by palatal flap elevation. These requirements linked the assessment of bone attachment sites with the tissue requirements of both flaps.

Failed oronasal fistula repairs may result in additional tissue loss and scarring, potentially complicating subsequent reconstruction [11,13]. In this dog, that concern and the documented periodontal tissue destruction led the surgeon to favor double-layer closure with the aim of achieving a secure initial repair. Available palatal and buccal tissues permitted an inverted palatal layer and oral coverage of both the repair and the palatal donor site. Securing the inverted flap presented a separate concern, as gingival recession and tissue loss limited the buccal gingival recipient margin available for attachment. Transosseous suture fixation to residual buccal alveolar bone was therefore selected to maintain flap apposition without relying solely on that margin.

Imaging and direct operative inspection supported this assessment at different stages. CT has been used to characterize periodontal lesions and oronasal defects in dogs, including the extent of bone loss and associated dental involvement [14,15,16]. In the present case, radiographs and conventional CT demonstrated loss of the osseous partition together with residual peripheral bone. Actual suture sites were selected by direct inspection after extraction and debridement. Imaging therefore identified potentially usable attachment regions, while accessibility and suitability for suture passage were assessed intraoperatively. This sequence illustrates the relevance of examining both the extent of bone loss beneath the fistula and the remaining anatomy at its margins.

Successful use of bone attachment also required the palatal flap to reach the selected sites without excessive tension. Preservation of vascular supply, careful tissue handling, and adequate mobilization remain fundamental principles of oral flap repair [6,8]. Preplacement of the four transosseous sutures allowed apposition and tension to be assessed, with further mobilization performed before tying. This provided an opportunity to adjust flap reach before completing fixation. Flap dimensions and displacement are also relevant considerations. A retrospective pilot study associated greater relative palatal flap area and increased flap-apex travel with failure, although these exploratory findings arose from a heterogeneous population dominated by neoplastic defects and do not establish selection thresholds for periodontal reconstruction [13]. They nevertheless support consideration of tissue requirements and displacement alongside the availability of attachment sites.

The role of transosseous suture fixation can also be considered in relation to adjunctive reconstructive materials. Auricular cartilage autografts and flexible bone membranes have been used in canine oronasal fistula repair [17,18]. More recently, collagen-membrane placement beneath the flap suture line was described in six dogs with eight periodontal fistula sites, including sites with deficient buccal gingiva, limited alveolar support, or reduced tissue mobility. The membrane was intended to provide adjunctive support, while closure remained dependent on appropriate flap design and tension release [19]. In the present repair, transosseous sutures supplied attachment for the inner flap, and the local tissues formed the oral–nasal barrier and oral covering. The sutures did not reconstruct the missing osseous floor. Flap attachment, tissue coverage, and support beneath a suture line therefore represent related but distinct components of reconstruction. Healing without an interpositional material demonstrates feasibility in this patient, while differences in defects and flap configurations preclude conclusions about the necessity or comparative benefit of adjunctive materials.

Management of the affected periodontal region was another component of reconstruction. Dental preparation and treatment of diseased teeth are integral to the management of periodontal oronasal fistulas [5,8]. Staged double-layer closure has been reported with selective maxillary extractions performed 4–8 weeks before reconstruction [20]. In the present case, scaling preceded repair by 24 days, whereas adjacent teeth were extracted during definitive reconstruction. This interval therefore differed from a period of extraction-site healing before flap surgery. Changes in tissue condition after scaling were not quantified, preventing assessment of the interval’s contribution to healing or definition of an optimal delay. Likewise, the recorded pre-extraction dimensions describe the initial opening and should be distinguished from the tissue requirements after extraction and debridement. The remaining teeth were retained at the owner’s request after discussion of the need for further periodontal assessment and possible additional extractions; this treatment choice should not be interpreted as evidence that the retained teeth required no further treatment.

The late reassessment provided an opportunity to evaluate maintenance of closure beyond early wound healing and resolution of nasal signs. At 555 days, visual oral examination showed an intact repair, and CT obtained for an unrelated otologic indication was consistent with soft-tissue coverage at the previous fistula site. The assessments provided complementary information: oral examination evaluated the visible closure, while CT assessed the reconstructed region in cross-section. Their concordance supports long-term maintenance of oral–nasal separation in this dog. However, the late assessment did not allow separate evaluation of the two original flap layers.

This report is limited by its single-case design and the concurrent use of extraction, margin preparation, transosseous suture fixation, double-layer coverage, and postoperative care. The contribution of transosseous suture fixation cannot be evaluated independently, and a similar outcome with a single flap or soft-tissue attachment cannot be excluded. Recipient-tissue quality and closure tension were assessed clinically, and fixation strength was not measured. Final prepared-defect dimensions and quantitative measurements of residual bone were unavailable, limiting anatomical comparisons; the four attachment sites reflected the anatomy encountered in this patient. Tissue changes during the 24-day interval between scaling and repair were not quantified. The absence of intraoral dental radiographs and detailed tooth-specific periodontal records limited retrospective assessment of disease severity and treatment needs in the retained dentition. Maintained fistula closure does not establish periodontal disease control in the remaining teeth. Follow-up consisted of discrete examinations. The favorable outcome therefore pertains to the completed reconstruction.

## 4. Conclusions

In this dog, an inverted palatal mucoperiosteal flap anchored to residual buccal alveolar bone and covered with a buccal advancement flap achieved closure of a periodontal oronasal fistula. Oral examination and CT at 555 days supported long-term maintenance of closure. This case illustrates how peripheral bone can provide flap attachment sites when gingival recipient margins are compromised, provided sufficient local tissue remains for coverage of both the fistula and palatal donor site without excessive tension. Further clinical evaluation is needed to define anatomical selection criteria and determine the contribution of transosseous suture fixation to repair outcomes.

## Data Availability

The data presented in this study are included in the article. Further inquiries can be directed to the corresponding author.

## Abbreviations

The following abbreviations are used in this manuscript:

CRP	C-reactive protein
CT	Computed tomography
POD	Postoperative day
